# Physiological relevance of covalent protein modification by dietary isothiocyanates

**DOI:** 10.3164/jcbn.17-91

**Published:** 2017-12-12

**Authors:** Toshiyuki Nakamura, Naomi Abe-Kanoh, Yoshimasa Nakamura

**Affiliations:** 1 Graduate School of Environmental and Life Science, Okayama University, 1-1-1 Tsushima-naka, Kita-ku, Okayama 700-8530, Japan; 2 Department of Food Science, Graduate School of Biomedical Sciences, Tokushima University, 3-18-15 Kuramoto-cho, Tokushima 770-8503, Japan

**Keywords:** isothiocyanates, cruciferous vegetables, covalent modification, protein targets

## Abstract

Isothiocyanates (ITCs), naturally occurring in abundance in cruciferous vegetables, are the most well-studied organosulfur compounds having an electrophilic reactivity. ITCs have been accepted as major ingredients of these vegetables that afford their health promoting potentials. ITCs are able to modulate protein functions related to drug-metabolizing enzymes, transporters, kinases and phosphatases, etc. One of the most important questions about the molecular basis for the health promoting effects of ITCs is how they modulate cellular target proteins. Although the molecular targets of ITCs remains to be validated, dietary modulation of the target proteins via covalent modification by ITCs should be one of the promising strategies for the protection of cells against oxidative and inflammatory damage. This review discusses the plausible target proteins of dietary ITCs with an emphasis on possible involvement of protein modification in their health promoting effects. The fundamental knowledge of ITCs is also included with consideration of the chemistry, intracellular behavior, and metabolism.

## Introduction

**Fig. 1:**
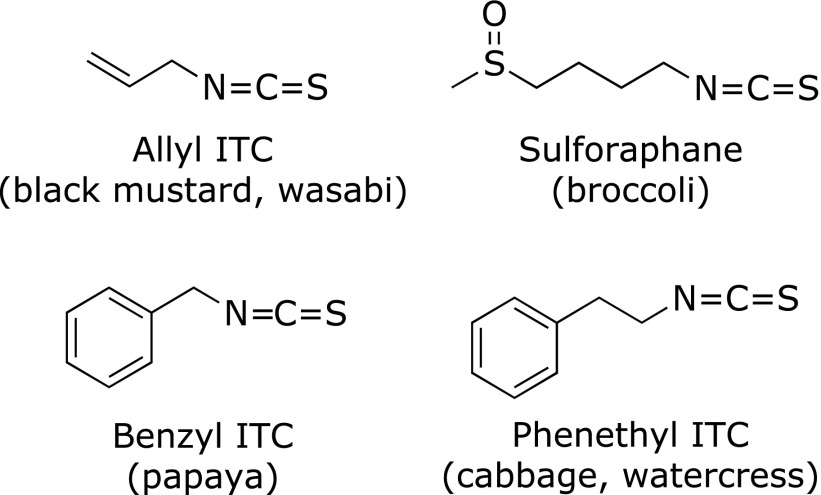
The chemical structures of the ITCs most extensively studied.

Isothiocyanates (ITCs) are one of the most famous organosulfur compounds having an electrophilic reactivity. ITCs have also been accepted as major ingredients of cruciferous vegetables to afford their health promoting and disease preventive potentials, such as anti-cancer, anti-inflammatory, and antioxidative effects.^(1)^ They are stored as glucosinolates (GLs; β-thioglucoside-*N*-hydrosulfates) in plants and are released when the plant tissue is damaged. There are over 120 GLs in various plants, each yielding the corresponding ITCs.^(2)^ Most GL-containing genera are categorized within the Brassicaceae, Capparaceae, Caricaceae and Moringaceae; Brassicaceae alone contains more than 350 genera and 3,000 species.^(2)^ All the investigated Brassicaceae species are thought to have the ability to synthesize GLs, such as aliphatic, methylthioalkyl, aromatic and indole GLs.^(3)^ For example, allyl ITC (AITC) is ubiquitous in cruciferous plants as its GL, sinigrin; sulforaphane (SFN), one of the most famous ITCs, is particularly abundant in broccoli (*Brassica oleracea* L. var. *italica*) in the form of its GL, glucoraphanin; β-phenethyl ITC (PEITC) is, also a well-studied ITC, found as gluconasturtiin, abundant in water cress (*Nasturtium officinale* L.); and benzyl ITC (BITC) is formed from benzyl glucosinolate (glucotropaeolin) in papaya (*Carica papaya*) (Fig. 1). The health promoting properties of cruciferous vegetables might be attributable to their GLs, which are also responsible for their pungent odor and biting taste. However, since GLs themselves are much less bioactive than the corresponding ITCs and can be metabolized by the myrosinase (MYR, thioglucoside glucohydrolase)-like activity of the intestinal microflora in humans, ITCs are accepted as the main principles for the disease preventive potentials of cruciferous vegetables.^(1)^

Among the various biological activities, chemoprevention against chemical carcinogenesis in experimental animals and its underlying mechanisms have been most extensively studied. ITCs have the abilities to inhibit both the formation and development of cancer cells through multiple pathways; i.e., inhibition of carcinogen activating enzymes and induction of carcinogen detoxifying enzymes, induction of antiproliferation by inducing cell cycle arrest and apoptosis, inhibition of cancer cell invasion and metastasis, inhibition of angiogenesis, etc.^(1,4)^ Accordingly, ITCs are able to modulate protein functions related to carcinogenesis, including phase 1 (cytochrome P450) and 2 drug-metabolizing enzymes, drug transporters, cell cycle regulators, caspases, kinases and phosphatases, etc. In addition, most of the protein targets are shared by the different types of ITCs in spite of their various side chains.

The most important question about the molecular mechanisms for the disease preventing effects of ITCs is how they modulate the functions of cellular target proteins. It is also very interesting to understand why different ITCs share similar molecular mechanisms, even though a certain effect is responsible for the specific ITC.^(5)^ The common structural point of ITCs is the presence of an ITC (-N=C=S) group, the central carbon of which is electrophilic and thus participates in a chemical reaction by accepting an electron pair of a nucleophile. Although ITCs could directly bind to DNA and RNA as well as proteins, no detectable binding with DNA or RNA was observed in human lung cancer cells,^(6)^ suggesting that DNA or RNA is unlikely to be a major target of ITCs in cells. ITCs actually inhibit glyceraldehyde-3-phosphate dehydrogenase (GAPDH), a model protein having active thiol groups, whereas the non-electrophilic *O*-methylthiocarbamate does not.^(7)^ This structure-activity relationship has also been observed in some biological activities, including the suppression of the NADPH oxidase-dependent superoxide generation,^(7)^ induction of glutathione *S*-transferase (GST) activity,^(8)^ and modification of mitochondrial respiration.^(9)^ Similar to the ITC metabolism, the health promoting effects of ITCs are regarded to be initiated, at least partly, by direct reaction of the ITC group with the nucleophilic amino acid residues in the target proteins. The side chains of ITCs may play an additional role in the interaction with the targets and cellular accumulation, possibly by influencing the electron density, steric hindrance and lipophilicity. Although validation of the real molecular targets of ITCs remains a big issue, dietary modulation of the key regulatory factors directly via covalent modification by ITCs should be one of the promising strategies for disease prevention and health promotion. This review includes the fundamental knowledge of ITCs on their chemistry, metabolism, absorption, and intracellular behavior. This review also discusses the plausible molecular targets of ITCs with an emphasis on physiological relevance of protein modification to their health promoting effects.

## Decomposition, Metabolism and Cellular Behavior of Isothiocyanates

**Fig. 2:**
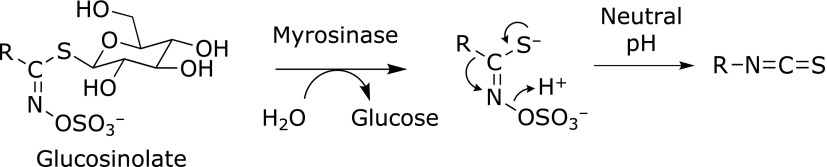
The conversion of GLs to ITCs. ITCs are formed by the hydrolysis of GLs by MYR in plants or intestinal microflora, followed by Lossen rearrangement under neutral conditions. At neutral pH, the major GL hydrolysis products are ITCs.

**Fig. 3:**
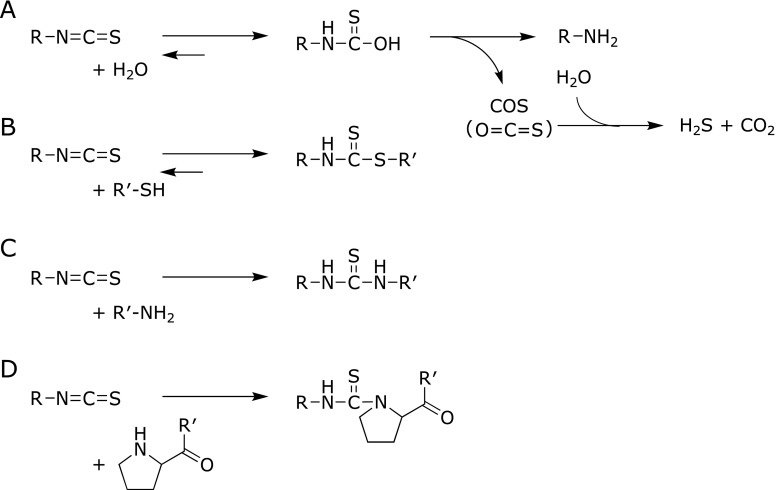
The schematic reactions between ITCs and nucleophiles. ITCs can react with water (A), thiol (B), primary amine (C) and secondary amine (D, proline) to yield monothiocarbamates, DTCs and TUs.

The conversion of GLs into ITCs is summarized in Fig. 2. The formation of ITCs is mediated by the hydrolysis of GLs by MYR in plants or intestinal microflora, followed by Lossen rearrangement under neutral conditions. At neutral pH, the major GL hydrolysis products are relatively stable ITCs, whereas a low pH prefers to produce nitriles.^(10)^ The main decomposition pathway of ITCs in neutral or alkaline media is the addition of a hydroxyl ion to the ITC group and formation of monothiocarbamates (Fig. 3A).^(11)^ Under a weakly alkaline or neutral condition, the amines and carbonyl sulfide (COS, O=C=S) are formed from the monothiocarbamates, the latter of which is further converted into hydrogen sulfide and carbon dioxide. The formed amines can further react with ITCs to yield thioureas (TUs), as shown in Fig. 3C. In addition, the rearrangement of ITCs into thiocyanates occurs during the GL metabolism or storage in aqueous solutions.^(11)^ Reduced glutathione (GSH), the most abundant cellular molecule carrying a thiol group (0.5–10 mM in cells), is a primary target for the conjugation of ITCs, which results in the formation of dithiocarbamates (DTCs) (Fig. 3B). In the cells, the DTC formation with GSH is enhanced by GST.^(12)^ ITCs also bind to cysteine sulfhydryl groups in proteins directly or via thiol exchange reactions. DTCs exist in equilibrium with the free form, dependent on the chemical characteristics of the sulfhydryl groups and ITCs, such as the p*K*a and electrophilicity, respectively.^(13)^ In addition to the cysteine-DTC formation, ITCs are able to react with amine groups in proteins, such as the α-amino groups in the N-terminal residues, ε-amino groups in the lysine residues and secondary amine groups in the N-terminal proline (Pro1) residues with the lower p*K*a to form stable TUs (Fig. 3C and D). The TU formation with amino acids is irreversible, but the rate of the reaction is much less than that of the DTC formation.^(14)^

**Fig. 4:**
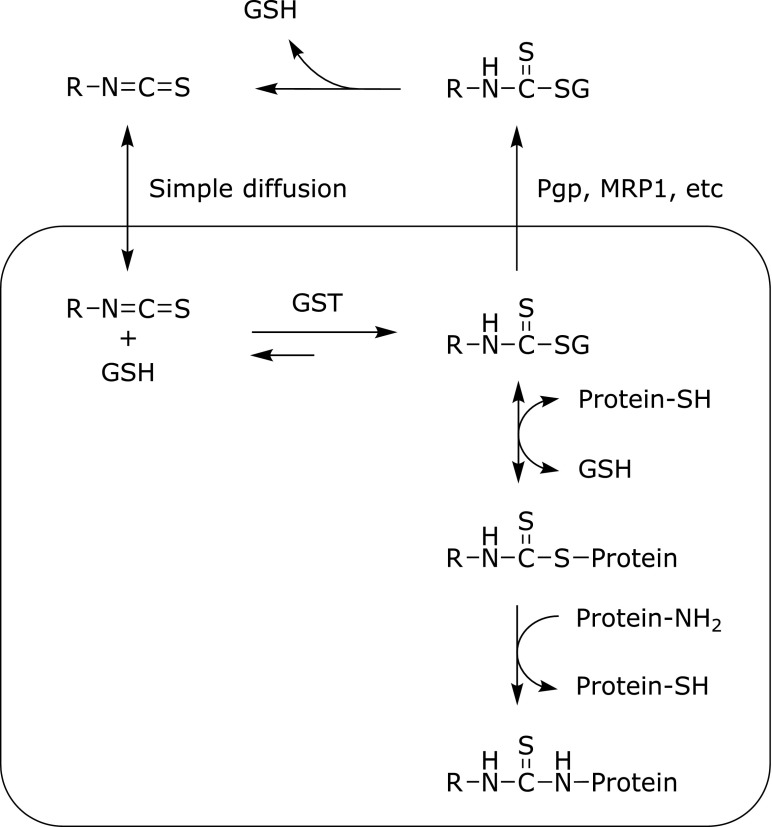
The cellular behaviors of ITCs. ITCs accumulate through simple diffusion and conjugation with GSH. The GSH-DTCs are excreted via membrane drug transporters. The GSH-DTCs also modify sulfhydryls or amines of cellular proteins by exchange reactions. The GSH-DTCs are exported through membrane drug transporters, such as multidrug resistance associated protein-1 (MRP1) and P-glycoprotein (Pgp).

The intracellular behavior of ITCs is well understood as shown in Fig. 4. The exposure of cells to ITCs leads to the rapid and high intracellular accumulation mainly through simple diffusion. The GSH conjugation by GST is also the major driving force for ITC accumulation by means of the enhanced intracellular trapping that prevents from diffusive efflux.^(15)^ When murine hepatoma Hepa1c1c7 cells were treated with 10 µM BITC or 50 µM SFN for 30 min, the total intracellular levels (ITC + DTCs) were estimated at 1.9 and 5.1 mM, respectively.^(16)^ Although the GSH-DTCs are initially predominant intracellular metabolites 30 min after the ITC treatment, their increasing amount in the culture medium and the shift of the binding toward cellular proteins were observed in the later period.^(6)^ Intracellularly accumulated ITCs are mainly exported as the GSH-DTCs, possibly through membrane drug transporters, such as multidrug resistance associated protein-1 and P-glycoprotein.^(17)^ Since the covalent conjugation with GSH is reversible, the GSH-DTCs can undergo either dissociation or replacement reactions with proteins.^(18)^ Intracellularly, ITCs might also be liberated from the DTCs and then react with the amine groups of proteins.^(19)^

**Fig. 5:**
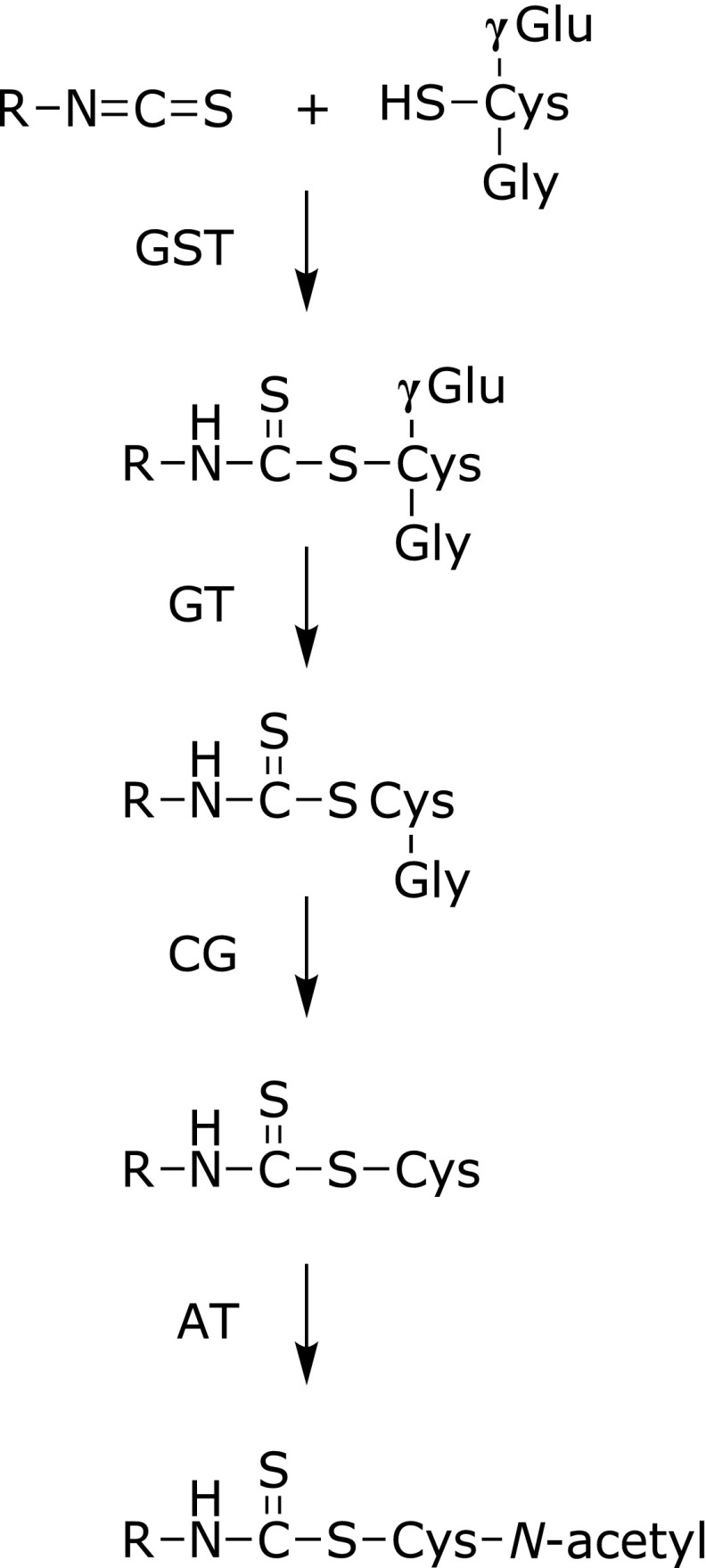
The mercapturic acid pathway-dependent metabolism of ITCs. ITCs initially react with GSH to form the GSH-DTCs, which are enhanced by GSTs. The conjugates undergo further enzymatic modifications; γ-glutamyltranspeptidase (GT) converts the GSH-ITCs into cysteinylglycine-DTCs, then cysteinylglycinase (CG) converts cysteinylglycine-DTCs into cysteine-DTCs, and *N*-acetyltransferase (AT) converts cysteine-DTCs into NAC-DTC conjugates.

The ITCs digested and absorbed in the body are predominantly metabolized via the mercapturic acid pathway (Fig. 5).^(20)^ GST isozyme polymorphisms have a significant impact on the overall ITC metabolism, because the first reaction of the ITC metabolism is GSH conjugation catalyzed by GSTs in the digestive tract and liver. The DTCs of GSH are further converted into those with cysteinylglycine, cysteine and *N*-acetylcysteine (NAC) via the mercapturic acid pathway in the kidneys, then excreted in the urine. Therefore, the uric level of the NAC-DTCs serves as a major metabolic marker of ITCs or the digested GLs.

## Analytical Methods for Detection of ITCs

The most frequently used detection method for ITCs or their DTCs is the cyclocondensation assay.^(21,22)^ This assay utilizes 1,2-benzenedithiol as the vicinal dithiol reagent and spectroscopically measures the reaction product, 1,3-benzodithiole-2-thione. However, this method cannot identify the tested ITCs, because the reaction with 1,2-benzenedithiol is dependent only on the ITC moiety and independent of the side chains. Numerous studies of the individual ITCs have used more strict analytical instruments, such as high performance liquid chromatography (HPLC) and gas chromatography (GC) often with mass spectrometry (MS) (Table 1). The ITCs derived from GLs by MYR have been classically measured by GC or GC-MS analysis.^(23–25)^ HPLC with MS(/MS) (LC-MS, LC-MS/MS), especially quadrupole (Q) or ion trap MS with electrospray ionization (ESI), has frequently been used as an analytical tool for the ITCs and their metabolites from *in vivo* samples, such as blood plasma, urine, liver, kidney and lung.^(26–38)^ The adduction between ITCs and proteins has also been investigated using MS, such as matrix assisted laser desorption ionization-time of flight (MALDI-TOF) and ESI-Q-TOF after enzymatic digestion of the tested proteins.^(39,40)^ In addition, specific monoclonal antibodies against the AITC-, BITC-, or 6-methylsulfinylhexyl ITC (6-MSITC)-lysine adduct have been developed.^(41)^ Using these antibodies, the ITC-lysine adduct formation was confirmed in the reaction mixture of ITCs and bovine serum albumin (BSA) by an enzyme-linked immunosorbent assay. In any case, the instrumental and immunochemical methods may be essential tools to understand the disposition and target molecules of the ITCs and their metabolites.

## Potential Targets of ITCs and Their Physiological Relevance

### Keap1

**Fig. 6:**
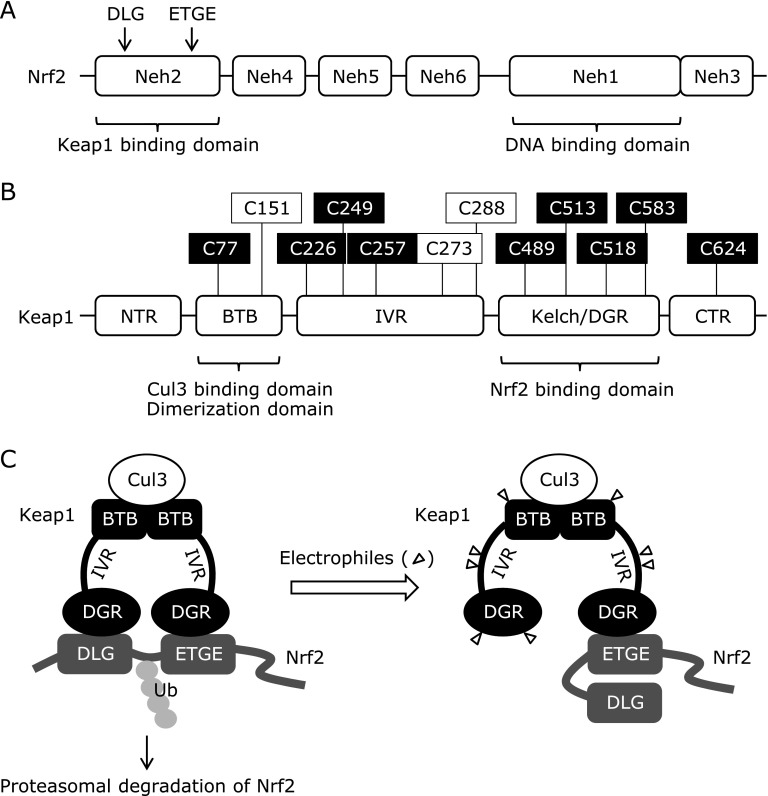
The structure of Nrf2 and Keap1. (A) Nrf2 has six Nrf2-ECH homology (Neh) domains. (B) Keap 1 consists of five domains, such as an N-terminal domain, a BTB (Broad complex/Tramtrack/Bric-a-brac) domain, a central linker domain [intervening region (IVR)], a Kelch repeat [double glycine-repeat (DGR)] domain and a C-terminal domain. Chemically reactive cysteines with SFN are illustrated in white font on a black ground. Functionally important cysteines are in black font on a white ground. (C) Nrf2 interacts with the Keap1 dimer at the DLG motif and the ETGE motif in the Neh2 domain to form the Keap1-Nrf2-Cul3 complex under basal conditions. Triangles represent electrophiles.

Among the plausible protein targets for ITCs, the cysteine-rich protein Keap1 [Kelch-like erythroid cell-derived protein with CNC homology (ECH)-associated protein 1], has been most extensively studied as a negative regulator of nuclear factor-erythroid 2 p45-related factor 2 (Nrf2) (Fig. 6). The Keap1-Nrf2 system plays a major role in the transcriptional regulation of various cytoprotective genes in response to electrophiles like ITCs as well as oxidative stress. Not only SFN, but also ITCs including BITC, PEITC and 6-MSITC, are able to up-regulate the Nrf2-targeted gene expression.^(8,42–45)^ Nrf2, as a heterodimer with small Maf, controls the gene transcription through the binding to the antioxidant response element (ARE) in the enhancer regions of its target genes. Among the six Nrf2-ECH homology (Neh) domains in Nrf2, the Neh2 domain contains two Keap1-binding sites, the DLG motif and the ETGE motif. Keap1 has five domains, such as an N-terminal domain, a BTB (Broad complex/Tramtrack/Bric-a-brac) domain, a central linker domain [intervening region (IVR)], a Kelch repeat [double glycine-repeat (DGR)] domain and a C-terminal domain.^(46)^ The BTB domain provides the dimer structure of Keap1 and binds to the ubiquitin E3 ligase Cullin3 (Cul3).^(47)^ The DGR domain is bound to the Neh2 domain in Nrf2 and to the actin,^(48)^ which contributes to the cytoplasmic sequestration of Nrf2. Under basal conditions, Nrf2 interacts with the Keap1 dimer at the ETGE motif and the DLG motif in the Neh2 domain to form the Keap1-Nrf2-Cul3 complex, which keeps Nrf2 at a low level via ubiquitin-proteasome degradation.^(49–51)^ Thiol modification by electrophiles results in structural changes in the complex. Although the conformational changes are not sufficient to release Nrf2 from Keap1 due to high affinity binding of the ETGE motif, they result in misalignment of the lysine residues between the DLG and ETGE motifs and disruption of the ubiquitination and proteasomal degradation of Nrf2.^(52)^

Zhang and Hannick ^(53)^ identified two cysteine residues in Keap1, Cys273 and Cys288 as critical targets for the Keap1-dependent ubiquitination and degradation of Nrf2. They also identified Cys151 as a SFN target for inhibition of the Keap1-dependent degradation of Nrf2. Several cysteine residues in Keap1 have been identified as the potential ITC binding sites using an *in vitro* ectopic expression system or overexpression experiments. SFN is able to modify 25 of the 27 cysteines of Keap1 in a concentration-dependent manner.^(46)^ Predominant SFN targeting sites in Keap1 were suggested; Cys77 in BTB domain, Cys226, Cys249 and Cys257 in central linker domain, Cys489, Cys513, Cys518 and Cys583 in Kelch repeat domain, and Cys624 in C-terminal domain. Since not only the Keap1 targeting selectivity, but also the ubiquitination switching inducibility varies with the electrophiles tested,^(46)^ further studies are needed to validate the actual functions of the cysteine residues modified by ITCs. Although ITCs are believed to activate the Nrf2 pathway through direct binding to Keap1, many other factors than Keap1 have also been postulated to regulate the Nrf2 pathway, including protein kinases, transcription factors and epigenetic modifications.^(54)^ Moreover, we have recently reported that BITC activates the Nrf2-dependent transcription via a Keap1-independent and phosphatidylinositide 3-kinase (PI3K)- and autophagy-dependent pathway.^(55)^ Future efforts will be concerned with the identification of other ITC targets activating the Nrf2 signaling than Keap1.

### Tubulin

Microtubules are the key components of the cytoskeleton that are responsible for many cellular processes including mitosis, cytokinesis, organization of the intracellular structure, intracellular traffic and cell motility. They are the tube-shaped dynamic polymers composed of α- and β-tubulin heterodimers. During mitosis, microtubules form the mitotic spindle through the formation of a bipolar array of microtubules emanating from the centrosomes.^(56)^ The mitotic spindle ensures that the replicated chromosomes equally separate at the end of the mitotic phase into two daughter cells. When the dynamics of the mitotic spindle are compromised, mitotic delaying or arrest occurs at the metaphase-anaphase transition, eventually leading to apoptosis.^(57)^ Therefore, microtubules are considered as one of the best therapeutic targets for rapidly proliferating cancer cells. Tubulin-binding agents (or microtubule-targeting agents), such as the taxanes that stabilize microtubules and the vinca alkaloids that destabilize them, are widely used as anti-cancer agents.^(58)^

ITCs have been shown to inhibit cell proliferation by inducing cell cycle arrest and apoptosis in various cancer cells.^(5,59–66)^ The antiproliferative effect of ITCs in human non-small lung cancer A549 cells followed the order of BITC > PEITC > SFN.^(39)^ The same order of potency is found in the binding affinities of ITCs to tubulin, the inhibition of tubulin polymerization *in vitro*, the disruption of microtubule networks, the induction of tubulin-containing protein aggregates, and degradation of tubulin through ubiquitin-proteasome system,^(39,67)^ strongly suggesting the physiological relevance of ITC binding to tubulin. Furthermore, *N*-methyl phenethylamine (NMPEA), a structural analog of PEITC without the ITC functional group, has no binding affinity for tubulin, suggesting that the ITC functionality is essential for the binding. Mi *et al.*^(39)^ identified α- and β-tubulin as ITC-binding targets to trigger cell cycle arrest and apoptosis induction using radioisotope-labeled ITCs. This is the first *in vivo* evidence of binding between ITC and an intracellular human protein. They also showed that ITCs having different side chains, such as BITC, PEITC and SFN, are able to bind at the same cysteine residue, Cys303, in the purified porcine β-tubulin.^(39)^

### Proteasome

The ubiquitin proteasome system, an essential and highly regulated mechanism to control intracellular protein degradation and turnover, plays important roles in various fundamental cellular processes, such as the regulation of cell cycle progression, division, apoptosis, cell trafficking, and the modulation of the immune and inflammatory responses. A recent proteomics study identified proteasome as a potential ITC-binding target.^(68)^ BITC and PEITC significantly inhibited both the 26S and 20S proteasome activities, possibly through the direct ITC binding, whereas NMPEA did not inhibit the proteasome activity under the same conditions,^(69)^ suggesting that the ITC functional group is essential for the ITC binding to proteasome like Keap1 and tubulin. The potency of the proteasome inhibition correlates with the rapid accumulation of p53 (tumor suppressor) and IκB (NF-κB inhibitor).^(69)^ Furthermore, BITC and PEITC significantly suppressed the cell proliferation of multiple myeloma, a type of blood cancer that is sensitive to proteasome inhibition, through induction of G_2_/M arrest and apoptosis.^(69)^ These findings indicate that ITCs exert a physiological activity through the dysfunction of proteasome by direct binding.

### TRPA1

Transient receptor potential cation channel, subfamily A, member 1 (TRPA1) is also known to activate by various pungent compounds including ITCs. TRPA1 plays a physiological role not only in thermosensing, but also thermogenesis and energy expenditure.^(70)^ A study using the TRPA1-expressing HEK cells demonstrated that the channel was activated by covalent modification of AITC to the cysteine residues.^(71)^ Of the 31 cysteine mutations, the triple mutant at Cys415, Cys422 and Cys622 showed no detectable calcium influx in response to AITC. Another study using cysteine substitutions at 13 of 21 positions that are invariant among human, rat, and mouse TRPA1 sequences indicated that the TRPA1 channel is activated by AITC, possibly through its interaction with three cysteine residues (Cys619, Cys639 and Cys663) in the cytoplasmic N-terminal tail of the channel.^(72)^ In addition to the cysteine residues, a lysine residue (Lys708), at least in part, contributed to the activation of TRPA1 by ITCs.^(72)^

### MIF

Macrophage migration inhibitory factor (MIF) is a homotrimeric multifunctional proinflammatory cytokine that has been implicated in the pathogenesis of many inflammatory and autoimmune diseases.^(73)^ It is also a tautomerase with a catalytically-active Pro1, even though a defined physiological substrate remains uncovered. ITCs, including BITC and PEITC, are reported to irreversibly inhibit the tautomerase activity of MIF through covalent binding to the Pro1 residue.^(74–76)^ Although MIF contains many cysteine residues, PEITC exclusively modified Pro1 and formed the TU adducts.^(75)^ This is probably because the Pro1 residue displays a low p*K*a (around 5.6–6).^(77)^ Functional changes induced by ITCs, including inhibition of the MIF tautomerase activity and interference with binding of MIF to its receptor CD7433, may contribute to the anti-inflammatory property of ITCs. The six ITC analogues, including AITC, BITC and PEITC, are able to modify the proline residue of the recombinant MIF with inhibition of the MIF tautomerase activity.^(76)^ Furthermore, Miyoshi *et al.*^(40)^ recently developed a novel method to identify the target molecules of the aromatic ITCs. This analytical method is based on monitoring a pattern of mass differences between BITC and PEITC (D = 14.01565). Using this method, they also identified MIF and GSH as plausible targets for the aromatic ITCs in human colon cancer HCT116 cells.^(40)^

### Others

Biochemical experiments using a biotinylated ITC probe indicated that ITCs inhibit the enzymatic activity of mitogen activated protein kinase/extracellular signal-regulated kinase kinase kinase 1 (MEKK1), possibly through the covalent modification of Cys1238 of MEKK1 with the ITC.^(78)^ Shibata *et al.*^(79)^ have also identified five proteins, i.e., Hsp90β, Hsp60, α-tubulin, β-actin, and GAPDH, as the ITC targets using alkynylated 6-MSITC and click chemistry. Some ITCs inactivate protein tyrosine phosphatase 1B (PTP1B) by covalent modification of the active site cysteine residue, Cys215.^(80)^ The PTP1B inhibition may play an important role in the modulation of the insulin receptor/PI3K/Akt survival pathway by BITC.^(66)^ In addition, the Cys112 of GSTA1, playing a key role in the catalytic activity, has been identified as a covalent modification target of PEITC by high-resolution MS.^(81)^

Besides the cysteine residues, ITCs are well-known to react with amine groups in proteins to form very stable TUs under alkaline conditions. Edman degradation, a representative method of the amino acid sequencing of polypeptides, is based on this principle.^(82)^ It should be noted that AITC is able to react with lysine residues in albumin as well as in lysine derivatives under neutral conditions.^(19)^ The AITC-lysine TU adduct in albumin was also detected in the reaction mixture of albumin and the AITC-NAC DTC, suggesting that the ITCs dissociated from the DTCs in proteins might react with ε-amino groups in the lysine residues.^(19)^ Consistently, covalent modification of Lys235, Lys437 and Lys548 in BSA by ITCs is quite probable, while the ITC-Cys58 adduct was not detected.^(83)^ Furthermore, the ITC-lysine TUs in albumin and hemoglobin have been identified in human plasma.^(84)^ The LC-MS/MS analyses of the digested proteins demonstrated that the TU adducts were detectable 1 day after consumption of ITC-containing foods, and that a half-life of the albumin adduct was 21–23 days.^(84)^ These results suggested that the ITC-lysine TUs in albumin and hemoglobin are very stable metabolic markers in human plasma after cruciferous vegetable intake.

Additionally, many other proteins have been identified as the binding targets of ITCs by a recent proteomics analysis; for example, actin, vimentin, thioredoxin, glutaredoxin-1, ubiquitin carboxyl-terminal hydrolase isozymes, heat shock proteins, NADH dehydrogenase 1, ATP synthase, cytochrome c oxidase copper chaperone, 14-3-3 protein, GSTP1, etc.^(68)^ However, these include several proteins abundantly existing in cells, such as actin, tubulin and vimentin,^(85)^ presumably due to the low binding specificity of ITCs, which makes it difficult to find the molecular targets strongly associated with the physiological activity of ITCs. Therefore, the screening system of ITC targets based on the ITC-induced phenotype is required to decipher the physiological relevance of the ITC binding to each protein.

## Conclusion

Undoubtedly, ITCs possess the potential to exhibit various biological activities, possibly through the covalent modification of certain protein targets. Transcriptional induction of the Nrf2-dependent drug-metabolizing and cytoprotective genes partly, but substantially, contributes to their health promoting effects. It should be noted that non-specific protein modifications by electrophiles, including ITCs, cause mild proteostress and may contribute to cytoprotection.^(86)^ On the other hand, the administration of excessive amounts of electrophiles provokes harmful effects, such as an induction of cytotoxicity and inflammatory reaction.^(1)^ For example, both BITC and PEITC promote urinary bladder chemical carcinogenesis in rats, possibly through necrotic cytotoxicity by severe protein modification.^(87)^ Our previous studies on the toxic effects of higher doses of phenolic acids^(88)^ also support this idea. If the dose employed is higher than that required for health promotion, the benefitial effects of ITCs might not be entirely expected. It is also noteworthy that the Nrf2 activation in cancer cells can contribute to the resistance to chemotherapeutic drugs.^(89)^ Accordingly, we have more recently demonstrated that BITC activates not only the PI3K/Akt/FoxO1 survival pathway,^(66)^ but also the PI3K/autophagy/Nrf2 cytoprotection pathway in human colon cancer cells.^(55)^ Therefore, further studies not only on the more precise molecular targets for ITCs, but also on the optimization of their dose or dose scheme for clinical studies are essential.

## Figures and Tables

**Table 1:** Detection methods for analysis of ITCs and their metabolites

Method	ITCs	Detection	Sample	Reference
GC	A, SFN, PE, B, etc.	ITCs	plant	(24)
GC-MS	A, PE, B, etc.	ITCs	plant	(25)
HPLC-UV	B	ITCs, GLs	plant	(26)
HPLC-UV	A	DTCs	urine (rat)	(27)
HPLC-UV	A	DTCs	urine (human)	(28)
HPLC-UV	PE	DTCs	urine, feces, tissue (mouse)	(29)
LC-MS	SFN	DTCs	intestinal contents (human)	(30)
LC-MS/MS	SFN	ITCs, DTCs	plasma, urine, feces, tissue (mouse)	(31)
LC-MS/MS	SFN, etc.	ITCs, DTCs	plasma, tissue (mouse)	(32)
LC-MS/MS	SFN, etc.	ITCs, DTCs	plasma, urine, tissue (mouse)	(33)
LC-MS/MS	A, SFN, PE, B, etc.	DTCs	urine	(34)
LC-MS/MS	SFN	DTCs	urine (human)	(35)
LC-MS/MS	SFN	ITCs, DTCs	urine (human)	(36)
LC-MS/MS	SFN	ITCs, DTCs	plasma, intestinal perfusate (rat)	(37)
LC-MS/MS	A, SFN, PE, etc.	ITCs	plasma (human)	(38)

A, allyl ITC; SFN, sulforaphane; PE, β-phenethyl ITC; B, benzyl ITC; GLs, glucosinolates; DTCs, dithiocarbamates.
